# Occurrence of and Dietary Exposure to Toxic Element Impurities in Food Additives: A Probabilistic Exposure Assessment for the Belgian Population

**DOI:** 10.3390/foods15203734

**Published:** 2026-10-20

**Authors:** Céleste Vansnick, Karlien Cheyns, Daniela Montalvo, Nadia Waegeneers

**Affiliations:** 1 Risk and Health Impact Assessment, Sciensano, 1050 Brussels, Belgium; 2 Trace Elements and Nanomaterials, Sciensano, 3080 Tervuren, Belgium; karlien.cheyns@vito.be (K.C.); daniela.montalvogrijalva@sciensano.be (D.M.); 3 Flemish Institute for Technological Research (VITO), 2400 Mol, Belgium

**Keywords:** food additives, toxic elements, impurities, dietary exposure, Monte Carlo simulation

## Abstract

Toxic element impurities in food additives may contribute to the dietary exposure to arsenic (As), cadmium (Cd), lead (Pb), and mercury (Hg). This study aimed to quantify these toxic elements in selected food additives available on the Belgian market and to assess their contribution to dietary exposure and associated health risks. Seventeen food additives were analyzed using ICP-MS/MS, direct mercury analysis, and LC-ICP-MS. Dietary exposure to toxic elements originating from food additives was estimated for different age groups using probabilistic modeling based on occurrence data and EFSA intake estimates for the food additives. The highest element concentrations were measured in algae-based additives, notably sodium alginate and carrageenan. Arsenic speciation showed predominantly organic forms in sodium alginate, while carrageenan contained up to 32% inorganic arsenic. Mineral additives and pectin also contributed to toxic element occurrence, whereas additives produced via a purification process showed negligible levels. The exposure to Pb through the combined intake of multiple food additives may reach up to ~18% of total dietary Pb exposure in adults. For children, margins of exposure for Pb and inorganic As were low (≈2–6), indicating that a health concern cannot be excluded. Exposure to Cd and Hg remained well below health-based guidance values.

## 1. Introduction

Chronic exposure to toxic elements, particularly arsenic (As), cadmium (Cd), lead (Pb) and mercury (Hg), is recognized as a significant public health concern due to their well-documented adverse health effects [1,2,3,4]. While these toxic elements primarily enter the food chain through natural pathways such as soil–plant–animal transfer, they may also be introduced through industrial processes, including their presence as impurities in food additives [5,6]. This contamination pathway represents an additional exposure route that can contribute to the overall dietary burden of toxic elements in the final food products consumed by the public. In Europe, more than 300 food additives are authorized under Regulation (EC) No 1333/2008 [7]. Citric acid, lecithins, ascorbic acid and xanthan gum are among the most frequently occurring and most frequently consumed food additives in products across European markets [8,9]. The estimated daily consumption of food additives may span several orders of magnitude, with carminic acid estimates as low as 0.1 mg/kg body weight (bw) per day for Belgian adults and glycerol estimates as high as 240 mg/kg bw per day for Belgian children [10,11].

The European Union has established regulatory frameworks to address the concern about food additives impurities, with Commission Regulation (EU) No 231/2012 [12] setting specifications for food additives listed in Annexes II and III to Regulation (EC) No 1333/2008 [7]. These specifications include maximum permissible limits for toxic elements, typically in the order of mg/kg. This is in contrast with maximum levels for contaminants in food, typically in the order of µg/kg, regulated in Commission Regulation (EU) 2023/915 [13]. However, despite the existence of these limits, there is a notable lack of publicly available data on the actual concentrations of these toxic elements in commercially available food additives.

Recent evaluations by the European Food Safety Authority (EFSA) Panel on Food Additives and Flavourings (FAF) have highlighted this data gap as a critical concern. In their re-evaluation of several food additives, including E 300–302, E 401–404, E 407, E 410, E 412 and E 415, the panel concluded that potential exposure to As, Pb, Cd, and Hg resulting from consumption of these additives could be significant if present at the specification limits [14,15,16,17,18,19]. This assessment underscores the need for comprehensive analytical data to accurately evaluate the contribution of food additives to overall dietary exposure to these toxic elements.

The analytical determination of toxic elements in food matrices presents well-established methodologies, being inductively coupled plasma–mass spectrometry (ICP-MS) measurement following closed-microwave extraction, the standard approach for As, Cd, and Pb determination (EN 13805:2014 [20] and EN 15763:2010 [21]), while direct mercury analysis (DMA) is a robust alternative for Hg quantification (EN 13806-3:2025 [22]) [23]. However, the diverse physicochemical properties of food additives present unique analytical challenges that require method-specific optimization. Mineral food additives may exhibit poor solubility in conventional acid extraction systems, create matrix effects in ICP plasma, or generate spectral interferences. Conversely, certain organic food additives may be highly reactive, posing safety risks during mineralization procedures due to their potential for explosive reactions.

Given the absence of comprehensive data on toxic element concentrations in food additives and the need to understand their contribution to overall dietary exposure in Belgium, this study aims to systematically evaluate the levels of toxic elements in a representative selection of food additives. The objective is to focus on additives that may contribute most significantly to population exposure through either high consumption rates or elevated susceptibility to toxic element contamination. This research involves the selection of relevant food additives, optimization and validation of analytical methods tailored to specific additive matrices, and comprehensive analysis of multiple samples to provide robust concentration data for exposure assessment purposes.

## 2. Materials and Methods

### 2.1. Selection of Relevant Food Additives

The selection of food additives, relevant in terms of potential toxic element exposure, took into account their occurrence frequency in food items on the Belgian market (based on data from the major retailers), previous element occurrence data obtained from the Belgian Federal Agency for the Safety of the Food Chain (FASFC) (if available), the maximum permissible level for each element and food additive specified in Commission Regulation (EU) No 231/2012, and the estimated intake of the food additive for the Belgian population (if available). The estimated intake was either derived from previous EFSA re-evaluations (non-brand-loyal mean exposure scenarios), or other refined-tier assessments [24,25]. Additional parameters that were considered in the selection of the food additives were whether the food additive could also be used in food as a nutrient or as a carrier, and whether the permitted use level of the food additive is specified as “quantum satis” in one or more food categories (Regulation (EU) No 1333/2008).

The 22 food additives that were considered relevant in terms of potential toxic element exposure were carminic acid (E 120), calcium carbonate (E 170), ascorbic acid (E 300), sodium ascorbate (E 301), citric acid (E 330), sodium phosphates (E 339), sodium alginate (E 401), carrageenan (E 407), locust bean gum (E 410), guar gum (E 412), xanthan gum (E 415), glycerol (E 422), pectin (E 440), polyphosphates (E 452), cellulose (E 460), methyl cellulose (E 461), sodium carbonates (E 500), potassium chloride (E 508), calcium chloride (E 509), calcium sulphate (E 516), carnauba wax (E 903) and aspartame (E 951). The food additives sodium alginate, calcium chloride, methyl cellulose, calcium sulphate and carnauba wax were selected due to the absence of national monitoring data and the calculation of a high potential exposure to toxic elements based on the authorized levels for mainly Pb and As in these food additives. Carrageenan, pectin, carminic acid and calcium carbonate were selected because high As, Cd, Pb or Hg levels (≥0.5 mg/kg) were previously observed during FASFC monitoring campaigns. Locust bean gum and glycerol were mainly selected due to their high intake estimates (>100 mg/kg bw per day), resulting in high potential exposure estimates. For citric acid and sodium carbonates, no potential exposure to toxic elements could be calculated, as no intake estimates are available. They are, however, the two most frequently occurring food additives in food items on the Belgian market and were therefore included in the selection. The remaining food additives were mainly selected based on their high occurrence frequency in food items on the Belgian market, combined with moderately high intake estimates (15–55 mg/kg bw/day) and high authorized toxic element levels in the food additives.

### 2.2. Food Additive Sample Collection

Food additives were obtained from distinct sources, namely official control samples provided by the FASFC (43%), by purchasing samples from retailers specialized in culinary products (online shop or physical store) (21%), via online unspecialized distributors (30%), or via contacts with food additive producer associations (6%). For each selected food additive, the objective was to obtain at least five samples, representing different manufacturers or production batches. Upon receipt, samples were homogenized and stored under dry conditions pending analysis.

### 2.3. Standards and Chemicals

Bi-distilled water was prepared in-house using an Aquatron system (A4000D; Cole-Parmer, Vernon Hills, IL, USA). A multi-element stock solution containing, amongst others, As, Cd and Pb at 100 ± 0.2 mg/L, was purchased from Analytika (Prague, Czech Republic). Individual stock solutions of As(V) were obtained from Inorganic ventures (Christianburg, VA, USA) at 1000 ± 5 mg/L, stock solutions of Cd and Pb from PerkinElmer (Pure Plus, Springfield, IL, USA) in 2% HNO_3_ at 1001 ± 3 mg/L and 999 ± 5 mg/L, respectively. For the calibration curve, a multi-element stock solution was diluted with nitric acid (HNO_3_, 67–69% *w*/*w*, Pico-pure; Chem-Lab, Zedelgem, Belgium) and bi-distilled water to final element concentrations at 0 mg/L, 0.0005 mg/L, 0.001 mg/L, 0.002 mg/L, 0.005 mg/L and 0.010 mg/L in 4% *v*/*v* HNO_3_. Independent control standards of 0.005 mg/L in 4% *v*/*v* HNO_3_ were prepared similarly from the individual stock solutions. The aqua regia solution (a 1:3 (*v/v*) ratio of HNO_3_ and hydrochloric acid (HCl 37% *w*/*w*, Normapur; VWR, Radnor, PA, USA)) was prepared 24 h before use to allow the reaction to be completed. Internal standards of indium and scandium were obtained from Fluka Analytical (Honeywell Research Chemicals, Morris Plains, NJ, USA). Stock solutions were stored in the dark in a refrigerator to prevent decomposition or oxidation. The internal standard solution containing scandium and indium at a concentration of 0.2 mg/L was prepared from these two solutions and adjusted with HNO_3_ and bi-distilled water. The tuning solution was prepared from Agilent Tune solution for ICP-MS containing Ce, Co, Li, Ti and Y at 10 mg/L and 2% HNO_3_. This solution was adjusted with HNO_3_ and bi-distilled water to a 0.001 mg/L solution. A brown rice reference material (As: 0.044 ± 0.009 mg/kg; Cd 0.036 ± 0.008 mg/kg; Pb: 0.036 ± 0.009 mg/kg; mean ± expanded measurement uncertainty) was prepared in-house. For arsenic speciation analysis, a brown rice flour certified reference material with an inorganic arsenic (iAs) concentration of 0.298 ± 0.008 mg/kg (NMIJ 7532-a; National Metrology Institute of Japan, Tsukuba, Japan) was used.

### 2.4. Analysis of Toxic Elements in Food Additives

Samples (sample amounts ranged between 10 and 1000 g) were mixed prior to analysis to ensure their homogeneity. Liquid samples or fine powders were homogenized by shaking the container by hand or using a plastic spatula. One flaked sample was mixed with an analytical grinder (IKA A11, IKA, Staufen, Germany). Samples were transferred to smaller containers to avoid contamination from the packaging. The determination of Hg in food additives was performed by a direct mercury analysis system (DMA 80, Milestone, Sorisole, Italy). The food additive sample (0.05 or 0.025 ± 0.005 g) was accurately weighed directly into the sample boat before being placed on the built-in autosampler. The measurement was taken in duplicate if the concentration was below the limit of quantification (LOQ) and in triplicate if the value was above the LOQ. The total concentrations of As, Cd and Pb were determined by triple quadrupole inductively coupled plasma–mass spectrometry (ICP-MS/MS) (Agilent 8800, Agilent, Santa Clara, CA, USA) following microwave-assisted acid digestion (Mars 6, CEM, Stallings, NC, USA). The methods were developed in-house based on standard methods for foodstuffs (EN 13805:2014 [20]). Hereto, 0.250 ± 0.025 g of the food additive sample was accurately weighed in acid-washed Teflon digestion vessels (CEM, Stallings, NC, USA). After weighing, 4 mL of concentrated HNO_3_ was added to each vessel and allowed to react for at least 15 min, after which 4 mL of bi-distilled water was added. The method was adjusted for carbonates (bi-distilled water added prior to HNO_3_), calcium chloride (reaction time extended to at least 30 min), and calcium sulphate (10 mL aqua regia added instead of 4 mL HNO_3_). After this first reaction, the samples were heated in the microwave system to 180 °C in 20 min and maintained at that temperature for 30 min. The digested samples were diluted in bi-distilled water prior to ICP-MS/MS analysis (final dilution factor of 400; for calcium sulphate, 1000). Pb (*m*/*z* 208) and Cd (*m*/*z* 114) were measured in He collision mode, while As was measured in mass-shift mode after reaction with O_2_ (*m*/*z* 75–>91). The introduction mode (general-purpose or high-matrix introduction (HMI)) was optimized based on the matrix load of the food additive samples. Three independent replicates were analyzed per sample. Quality control was ensured through the use of internal standards (indium and scandium) and by analyzing an in-house reference material (brown rice) in triplicate within each sample series.

Inorganic As was measured in relevant samples, i.e., organic food additives with total As concentration above 0.1 mg/kg (9 samples in total). Inorganic As in the selected samples was determined by liquid chromatography (LC) ICP-MS (iCAP rQ, Thermo Scientific, Waltham, MA, USA). Samples (0.250 ± 0.025 g) were extracted in duplicate with 0.1 M HNO_3_ containing 3% hydrogen peroxide (H_2_O_2_) using a CEM MARS microwave system (90 °C, 1 h). After filtration (0.45 µm), chromatographic separation was performed on a Dionex IonPac AG7 guard column (2 × 50 mm) (Thermo Scientific, Waltham, MA, USA) and AS7 analytical column (2 × 250 mm) (Thermo Scientific, Waltham, MA, USA) at 40 °C, with the autosampler maintained at 4 °C. A 25 µL injection volume was used. The mobile phase consisted of (A) Milli-Q water and (B) 200 mM diammonium carbonate (NH4)_2_CO_3_, delivered at 0.5 mL min^−1^ with the following gradient: 0–2 min, 15% B; 2–5 min, 15–100% B; 5–6 min, 100% B; 6.1–10 min, 15% B.

Arsenic was monitored at m/z 75 (KED with He). Baseline separation of As(V) was achieved and quantification was performed by external calibration using species-specific standards. Inorganic As was reported as the sum of As(III) and As(V). For the remaining samples (inorganic additives or organic additives with total As concentration below 0.1 mg/kg), the iAs concentration used in the exposure calculation was assumed to be equal to the total As concentration. Likewise, for all samples, given the low concentrations of total Hg measured, the analysis of inorganic mercury (iHg) or methyl mercury (MeHg) was not performed. As a conservative approach, Hg concentrations used in the exposure calculation are equal to the total Hg concentration.

### 2.5. Method Validation

The measurement of Hg by direct mercury analysis has previously been validated in-house for food and mineral matrices and is accredited for food analyses according to the ISO 17025 standard (BELAC 081-TEST) [26]. The same counts for the analysis for inorganic As in the selected samples. Hence, no separate validation was required for the food additives. The measurement of As, Cd and Pb by ICP-MS/MS is based on an in-house validated method that has also been accredited for food analyses, but because of the method adaptations and the mineral matrix of some food additives, an additional validation was performed. Hereto, the organic food additives were classified into distinct groups based on their molecular structure. For each group of food additives, a model food additive was used to validate the analytical method, thereby limiting the number of required method validations. For the mineral food additives, a validation was performed for each matrix.

A variety of performance parameters was evaluated for method validation, including recovery, repeatability, reproducibility, LOQ, limit of detection (LOD = LOQ/3), and measurement uncertainty. These performance parameters were evaluated against established acceptance criteria for food contaminants (Commission Regulation (EC) No 333/2007) to ascertain the reliability of the method for the analysis of food additives. Recovery, repeatability and reproducibility were determined by spiking each model food additive at three different levels. Spike concentrations ranged from 0.10 mg/kg to 5.0 mg/kg. To determine LOQ and LOD, procedure blanks were analyzed under repeatability conditions [27].

### 2.6. Exposure Assessment

The aggregated exposure to Cd, Pb, iAs and Hg through the combined intake of selected food additives was estimated via probabilistic modeling. Food additives were included in the aggregated exposure assessment for an element if at least 80% of the analytical results for the food additive were above the LOD and at least 20% were above the LOQ. Food additive intake was modeled by applying a chosen probability distribution model (Supplementary Materials Table S1) to intake estimates previously derived by EFSA (non-brand-loyal mean and 95th percentile intake for different age groups in Belgium [10,11,15,16,17,19,28,29,30,31,32,33]). Toxic element impurity concentrations in food additives were also modeled using a selected probability distribution (Supplementary Materials Tables S2–S5). Impurity concentrations below the LOD were set equal to zero before the distribution modeling. For concentrations between the LOD and LOQ, measured values were retained. The probabilistic exposure assessment was carried out through Monte Carlo simulations. A random toxic element concentration in the food additive (*C_FA_*; in µg/kg) and a random intake of that food additive (*DI_FA_*; in mg/kg bw/day) (both derived from the probability distributions) were multiplied by each other, a process that was repeated for each food additive, and the obtained exposures were summed over all food additives to obtain the aggregated exposure (*EDI*) for an individual (in µg/kg bw/day):

(1) $$EDI=\sum_{\;FA=\;1}^{n}C_{FA}\cdot DI_{FA}/1000\;$$

This calculation was repeated multiple times to obtain a population of exposed individuals. Mean, median and high-level exposure data were derived from this population. The assessments were performed for 10,000 pseudo-individuals of children (3–10 years), adolescents (11–17 years), adults (18–64 years) and elderly (65+ years). The probability distribution modeling and probabilistic exposure assessment were conducted in the Open-Source RStudio integrated development environment for R (version 2023.12.1+402) using the R Stats package (version 4.2.3) and the truncnorm package (version 1.0-9). The scripts are made available at https://doi.org/10.5281/zenodo.22654960, accessed on 9 September 2026.

The aggregated exposure estimates were compared to benchmark dose lower confidence limits (BMDLs) for Pb and inorganic As, and to tolerable weekly intakes (TWIs) for Cd and Hg (Table 1). For Cd and Hg, the aggregated exposure estimates were multiplied by 7 to obtain weekly exposure estimates. For Pb and inorganic As, EFSA considered that a margin of exposure (MOE) approach was appropriate for risk characterization because iAs is a genotoxic carcinogen and there was no evidence for a threshold for critical Pb-induced effects [3,34]. The MOE is calculated as [35]:

(2) $$MOE\;=\;\frac{BMDL}{Estimated\;exposure}$$

To interpret the MOEs, the interpretation in the respective EFSA evaluations was followed [3,34]. For inorganic As, an MOE of 1 corresponds to a 5% increase in incidence of skin cancer relative to the background incidence and raises a health concern [34]. For Pb exposure in children, an MOE greater than 10 would be sufficient to ensure no appreciable risk of a clinical neurodevelopmental effect [3]. At MOEs between 1 and 10, the risk is likely to be low, but not so low as to be dismissed as having no potential concern. For Pb exposure in adults, there is no appreciable risk of clinically significant nephrotoxic or cardiovascular effects at an MOE of 10 or higher, and even at MOEs between 1 and 10, the risk would be very low.

## 3. Results

### 3.1. Collection of Relevant Food Additives

Out of the 22 selected food additives, 19 could be collected. However, two of them (sodium phosphate and cellulose) were excluded because only one sample of each could be obtained (Table 2). Challenges associated with sampling food additives included difficulty in identifying the producers of raw materials, the use of pre-blended food additive mixtures by the food industry, the lack of a food certificate, and high minimum purchase quantities for raw materials. Food additives that could not be collected for these reasons were polyphosphates, carnauba wax, and aspartame.

### 3.2. Analytical Method Performance

The performance parameters derived for the ICP-MS/MS analyses of food additives are provided in Table 3. The quantification limits were generally lower for the regular mode than for HMI mode. The pooled repeatability relative standard deviation (RSDr) ranged from 1.5% to 7% across the different toxic elements and food additives, while the pooled reproducibility (RSDrw) reached 12%. The Horwitz Ratio for repeatability ranged from 0.12 to 0.76 across the different food additive groups, toxic elements and spike levels, while the Horwitz Ratio for reproducibility ranged from 0.09 to 1.04. The acceptance criteria were hence fulfilled. No distinct differences could be observed among the different types of food additives. The apparent recoveries ranged from 90% to 110% across the different toxic elements and food additives, and the observed bias was not significant. Considering the different validation parameters, the expanded measurement uncertainty (U) ranged from 13.2% for Pb in calcium chloride to 33.3% for As in calcium sulphate.

### 3.3. Toxic Element Impurities in Food Additives

The methods were applied to determine toxic element impurities in the collected food additives. Mercury was not determined in all food additive samples due to the corrosive behavior of high salt matrices in the DMA. Measured concentrations of As, Cd, Pb and Hg are described in Table 4. The results show a general low toxic element level for ascorbic acid, sodium ascorbate, citric acid, guar gum, glycerol, methyl cellulose and sodium carbonate. All these additives have median concentrations <15 μg/kg for every toxic element analyzed. The median Pb concentration for ascorbic acid is <LOD, but one sample was measured at 245 μg/kg.

For carminic acid, calcium carbonate, sodium alginate, carrageenan, locust bean gum, xanthan gum, pectin, potassium chloride, calcium chloride and calcium sulphate, the toxic element concentration was elevated for at least one of the analyzed elements. The results for Hg are below or close to the LOQ (0.6 µg/kg), except for carminic acid, which has a median concentration of 16 μg/kg.

The results show significant variability between the different analyzed additives and between different distributors of the same additive: carrageenan has a median As concentration of 354 µg/kg, with a range from 54 µg/kg to 1516 µg/kg. Likewise, carrageenan has a median Cd concentration of 110 µg/kg, with a range from 30 µg/kg to 623 µg/kg. For Pb, one sample of sodium alginate was measured at 2147 µg/kg, while the median is at 187 µg/kg. High Pb values were also measured in single samples of pectin and calcium chloride.

### 3.4. Toxic Element Exposure Through the Combined Intake of Food Additives

The estimated mean aggregated exposure to Pb from the combined intake of the analyzed food additives is 0.033 µg/kg bw/day to 0.038 µg/kg bw/day for adult persons and the elderly (Table 5), leading to an MOE close to 20 in relation to the BMDL for nephrotoxic effects and an MOE near 40 in relation to the BMDL for cardiovascular effects [3]. Since the MOEs are above 10, no appreciable risk of clinically significant changes in the prevalence of chronic kidney disease or significant effects on systolic blood pressure are to be expected from food additive intake. EFSA estimated that the mean dietary exposure of Belgian adults was in the range of 0.2 to 0.5 µg/kg bw/day, which implies that the mean aggregated exposure from food additive intake may reach up to 18% of the mean dietary exposure for adults [39]. The estimated 95th percentile aggregated exposure to Pb from the combined intake of food additives reached 0.07 µg/kg bw/day for adult persons, resulting in MOEs of 10 and 20 for nephrotoxic and cardiovascular effects, respectively. For children, the mean aggregated exposure to Pb from combined food additive intake is estimated at 0.12 µg/kg bw/day, yielding an MOE of 4 for developmental neurotoxicity. Although the MOE is still above 1, and hence the risk is likely to be low, it cannot be dismissed as of no potential concern. The 95th percentile aggregated Pb exposure from food additive intake (0.23 µg/kg bw/day) results in an MOE of 2. The three main contributing food additives to Pb exposure are sodium alginate, pectin and calcium chloride (details in Supplementary Materials Table S6). Together, they account for 60 to 66% of the aggregated Pb exposure, depending on the age group. For children and the elderly, sodium alginate is the major contributor (31% and 26%, respectively), while for adolescents and adults, calcium chloride is the major contributor (35% and 28%, respectively).

To assess the aggregated exposure to iAs from the combined intake of food additives, it was assumed that total As equals the inorganic As fraction, unless speciation analysis was performed (sodium alginate and carrageenan). The estimated mean and 95th percentile aggregated exposures to inorganic As are 0.01 and 0.02 µg/kg bw/day, respectively, for adolescents and adults (including elderly people) (Table 5), which yields MOEs of 6 and 3, respectively, and cannot be dismissed as of no potential concern. For children, the estimated aggregated exposure from food additive intake is three times higher than that of adults. The dietary exposure to iAs was estimated by EFSA at 0.09–0.26 µg/kg bw/day for children and 0.04–0.11 µg/kg bw/day for adults, which implies that the mean aggregated exposure from food additive intake may contribute up to 33% to exposure. The three additives that mainly contribute to (inorganic) As exposure are calcium carbonate, carrageenan and calcium sulphate, which together account for 80% of the aggregated exposure (details in Supplementary Materials Table S7). Calcium sulphate accounts for half of this contribution. It should be noted that citric acid met the criteria for inclusion in the exposure calculations but could not be included due to the lack of available intake data.

The estimated mean aggregated exposure to Cd from the combined intake of food additives ranged from 0.05 µg/kg bw/week for adults and elderly people to 0.2 µg/kg bw/week for children (Table 6), which is low compared with the TWI of 2.5 µg/kg bw/week (2% and 7%, respectively). The estimated 95th percentile exposures are two to three times higher. Adding dietary exposure, calculated at a mean of 0.98 µg/kg bw/week and a 95th percentile of 2.02 µg/kg bw/week for adults [40], would not exceed the TWI for adolescents and adults; hence, health risks are not expected. For children, it is unclear whether the TWI would be exceeded when dietary exposure is added. Two food additives, calcium carbonate and carrageenan, account together for more than 80% of the aggregated exposure (details in Supplementary Materials Table S8). For children, carrageenan is the major contributor (47%), whereas for the other age groups, calcium carbonate is the major contributor (44% to 51%).

The estimated aggregated exposure to Hg from the combined intake of food additives (Table 6) was below 0.5% of the TWI (both for inorganic Hg and methyl mercury, if all Hg would have been present as iHg or MeHg, respectively), even at the 95th percentile exposure for children. It can hence be concluded that food additives do not contribute significantly to any Hg-induced health risks. Sodium alginate provided the largest contribution to Hg exposure for all age groups (23–30%), followed by carrageenan (15–20%) (details in Supplementary Materials Table S9).

## 4. Discussion

Among organic food additives, the highest toxic element concentrations were measured in algae-based polysaccharides. Sodium alginate (E 401) contained up to 685 µg/kg As, 9.3 µg/kg Cd, and 2147 µg/kg Pb, while carrageenan (E 407) showed concentrations of up to 1516 µg/kg As (including 152 µg/kg of iAs), 623 µg/kg Cd, and 282 µg/kg Pb. All measured values were compliant with the current specification limits established in EC 231/2012. Previous studies have reported elevated concentrations of As, Cd, and Pb in carrageenan samples, with values reaching 2040 µg/kg As, 1700 µg/kg Cd, and 2040 µg/kg Pb, varying with the harvest location [5,41]. Similar findings were considered during the 2018 EFSA re-evaluation of carrageenan in which interested parties submitted analytical data from ten non-consecutive production batches obtained from five manufacturers. Reported concentrations ranged from 0.03 to 1.50 mg/kg for arsenic, 0.02 to <2.00 mg/kg for lead, and 0.13 to <2.00 mg/kg for cadmium, and were below the limit of detection for mercury [17]. Likewise, during the EFSA re-evaluation of alginic acid and its salts (E 400–E 404), concentration data compiled from dozens of samples submitted by industry stakeholders indicated ranges of 0.1–2.2 mg/kg for arsenic, 0.1–0.5 mg/kg for lead, and 0.01–0.60 mg/kg for mercury [15]. In both re-evaluations, the EFSA panel concluded that the existing specification limits for toxic elements may not adequately reflect current manufacturing practices and recommended that the European Commission consider revising the specification limits for As, Cd, Pb and Hg in these food additives.

Sodium alginate and carrageenan are both polysaccharides of biological origin used as thickening and gelling agents extracted from brown or red seaweeds, respectively [15,17]. This algae origin is the cause of the elevated toxic element concentrations in the food additives, as algae are known to accumulate toxic elements [41,42,43]. However, the chemical speciation profile for As in algae is very diverse and the toxicity of As depends on the chemical species present [44,45,46]. Therefore, speciation analysis was performed for these algae-based food additives. These results show that As in sodium alginate (extracted from brown algae) is predominantly organic, with a maximum iAs concentration of 18 µg/kg. In contrast, iAs in carrageenan samples accounted for 10–32% of the total As content, with a maximum of 152 µg iAs/kg. The red algae *Kappaphycus alvarezii* (also known as *E. cottonii*) is one of the most important sources of commercial carrageenan [42,47] and inorganic arsenic has been identified before in *Kappaphycus alvarezii* in the range 57–202 µg/kg iAs. Cultivation methods could be optimized to minimize iAs uptake in these algae [48].

The other selected polysaccharide, pectin (E 440), can be extracted from residues of several plant-based food waste, usually citrus fruits or apples [49]. It is obtained by extraction in an aqueous medium of strains of appropriate edible plant material. This variable source can explain the wide range of toxic element concentrations (Cd < 2.3–521 µg/kg; Pb < 13.2–1019 µg/kg) measured in the seven different batches of pectin.

Limited toxic element contamination was detected in gum additives, with Pb concentrations reaching 228 µg/kg in locust bean gum (E 410) and 85 µg/kg in xanthan gum (E 415). The other detected toxic element concentrations in gums did not exceed 33 µg/kg. Locust bean gum and guar gum (E 412) are the ground endosperm of the carob tree or guar plant, respectively, which can take up toxic elements. Xanthan gum is produced by a culture fermentation of a carbohydrate with strains of *Xanthomonas campestris* and contamination depends on the raw materials used in the production process. Similar contamination levels were observed in a previous study by Azevedo et al. [5], where in guar gum samples, neither As nor Hg were detected (LOD 47 µg/kg and 9 µg/kg, respectively), while Cd and Pb were reported at 18 µg/kg and 28 µg/kg, respectively. In xanthan gum, Hg was detected at 87 µg/kg and Pb at 39 µg/kg. Similar conclusions can be drawn from the EFSA re-evaluations of locust bean gum, guar gum and xanthan gum, for which occurrence data submitted by industry generally showed concentrations of As, Cd, Pb, and Hg at levels below or only slightly above the limits of quantification. One exception is the measurement of Pb (21–770 µg/kg) in clarified locust bean gum [16,18,19].

Regarding the other organic food additives, toxic element contamination was also detected in carminic acid and carmines (aluminum lake form of carminic acid), both listed under E 120. Concentrations of Pb and As were detected up to 872 and 149 µg/kg (98 µg iAs/kg), respectively. This food additive has a biological origin (cochineal extract) and carminic acid has the ability to form chelates with toxic element ions (aluminum and calcium) to form carmines, which means it can also potentially bind with other heavy toxic elements [50]. No toxic elements were detected in either the cellulose (E 460) sample (only one sample was available) nor in the methyl cellulose food additives (E 461). The raw materials used for producing different types of celluloses are mainly wood chips. Microcrystalline cellulose (E 460(i)) is prepared by the controlled hydrolysis of highly purified α-cellulose, while powdered cellulose (E 460(ii)) is obtained by purification and mechanical disintegration of α-cellulose [32]. Methyl cellulose (E461) is a modified cellulose using sodium hydroxide and methyl chloride. The extensive purification processes employed in manufacturing these additives result in products with low toxic element content.

Apart from traces of As (max 9 µg/kg), no toxic elements were detected in the glycerol (E 422) samples. Glycerol can be produced by a variety of methods, as identified by EFSA [10,51]. However, extensive purification processes are performed to obtain food-grade glycerol, which accounts for the low toxic element content observed.

In the production of ascorbic acid (E 300), first sodium ascorbate (E 301) is produced by hydrogenation of D-glucose to D-sorbitol, microbiologically oxidized to L-sorbose, and converted through oxidation steps to 2-keto-L-gulonic acid, which is transformed to sodium ascorbate. The product is then precipitated, washed with methanol, purified, decolorized, and concentrated under vacuum before crystallization. Hereafter, ascorbic acid is produced by passing a solution of sodium ascorbate over cation exchange resin, resulting in non-detectable concentrations of toxic elements [14]. No toxic elements were detected in the ascorbic acid and sodium ascorbate samples, except for one ascorbic acid sample, where 245 µg/kg Pb was detected.

The main industrial production of citric acid (E 330) as a food additive uses fermentation by microorganisms, where species such as *Aspergillus niger* (fungi) and *Candida* (yeast) metabolize various carbon sources, including molasses and starch-based culture media. Following separation, purification, crystallization, and drying processes, the final powder product is obtained. Care should be taken as to which carbon resources are used for fermentation to avoid toxic element contamination, as citric acid has excellent metal ion chelating properties [52]. However, the downstream processes yield highly pure citric acid with no detected Pb, Cd and Hg, but trace As concentrations (up to 47 µg/kg) were detected in some samples.

Sampling of pure phosphate additives was difficult and only one sample of sodium phosphate (E 339) was obtained and analyzed. Only As (expected inorganic) was detected at a concentration of 62 µg/kg. Phosphoric acid can be produced cheaply by a wet process, in which the presence of toxic element impurities is unavoidable. However, for food purposes, phosphoric acid can also be derived via a thermal route in which elemental phosphorus is oxidized to diphosphorus pentaoxide (P_2_O_5_) and then hydrated to phosphoric acid, resulting in high-purity orthophosphoric acid, where only the impurity arsenic needs to be removed in an additional purification step [53,54].

Regarding mineral food additives, notable findings concern Cd and As concentrations in calcium carbonate (E 170), with Cd reaching a maximum of 471 µg/kg (median 207 µg/kg) and As reaching a maximum of 252 µg/kg (median 107 µg/kg). Elevated Pb concentrations were also observed in calcium chloride (E 509), with a maximum of 2252 µg/kg (median 1160 µg/kg). Notably, the highest measured concentration exceeded the EU specification limit of 2000 µg Pb/kg but the sample was compliant when the measurement uncertainty was considered. Data submitted by industry during EFSA evaluations reported comparable concentrations of toxic elements in calcium carbonate. Reported quantifiable concentrations ranged from 50 to 790 µg/kg for As, 30 to 400 µg/kg for Pb, 41 to 800 µg/kg for Cd, and 10 to 50 µg/kg for Hg. The Cd concentrations observed in the present study are therefore within the range previously reported by industry, whereas the measured Pb concentrations in the calcium carbonate samples exceeded the upper values reported in those datasets. It should be noted, however, that the industry data relate specifically to food additives manufactured for infant and young child nutrition [33].

For other mineral food additives, As was detected in calcium sulphate (E 516) at concentrations up to 575 µg/kg (median 277 µg/kg). The As concentrations are particularly significant, given that all arsenic in mineral food additives can be assumed to be present in its inorganic form [55]. An important consideration is that for mineral matrices, not all toxic elements are necessarily accessible to humans upon ingestion. Bioaccessibility refers to the fraction of a component that becomes soluble in the gastrointestinal tract, which differs from bioavailability, the fraction that ultimately reaches systemic circulation [56]. For mineral matrices, the critical consideration for risk assessment is not merely the total toxic element concentration, but rather the extent to which these toxic elements are bioaccessible in the gastrointestinal tract. Metal bioaccessibility has been extensively studied in the context of metal-contaminated soils [57] and should ideally be considered when performing exposure assessments of these toxic elements via food additives.

The uncertainties related to the toxic element impurity exposure estimates include those associated with food additive intake and those related to impurity concentrations in the food additives. Only 9 to 12 food additives were considered in the aggregated exposure estimates, while dozens are present on the market. This might lead to an underestimation of the aggregated exposure. The food additives were, however, selected based on their high potential relevance to toxic element exposure for the Belgian population, and of the 17 collected food additives, some already appeared to have low relevance for toxic element exposure. Trace element impurities were analyzed in only 5 to 10 items per food additive. Batch-to-batch variations in impurity concentrations could not always be considered, nor could the representativeness of the collected samples for the entire market be assessed, which may lead to large uncertainties in the exposure estimates. The food additive intake estimates retrieved from EFSA re-evaluations are considered an overestimation, as it was assumed that all products within a food category in which the additive is authorized contain it. To avoid propagation of uncertainty, only the non-brand-loyal intake estimates were considered in the exposure estimation. The probability distributions, both for the intake estimates and the toxic element occurrence estimates, are unknown, and the data to derive the distributions are limited (mean and 95th percentile food additive intake values, three to ten concentration data). The distribution models were selected using these limited data and expert judgment, and their suitability cannot be assessed statistically. The selection of the model can have a large impact on the exposure assessments and may lead to both over- and underestimations. In the calculations, it is assumed that the intake of food additives is independent, whereas in reality, co-occurrences can be expected. This may also lead to over- or underestimation of the aggregated exposure. Overall, the uncertainties associated with the aggregated exposure assessment are large, and it is unclear whether the exposure data are likely to be over- or underestimated.

## 5. Conclusions

This study demonstrates that toxic element impurities in food additives on the Belgian market are generally well below current EU specification limits. However, elevated concentrations were observed in certain additives sourced from organic and mineral origins, particularly algae-derived polysaccharides and some mineral additives. Although the contribution of these food additives to the exposure to Cd and Hg was negligible, food additives may contribute substantially to overall dietary exposure to Pb and inorganic As, especially in children. These findings support continued monitoring of toxic element impurities in food additives and provide evidence for ongoing evaluation of current specification limits.

## Figures and Tables

**Table 1:** Overview of health-based guidance values (HBGVs) used in the risk assessment. TWI = tolerable weekly intake, BMDL = benchmark dose lower confidence limit.

Element	Type of Reference Value	HBGV	Reference
Cd	TWI	2.5 µg/kg bw/week	[2]
Hg	TWI	iHg: 4 µg/kg bw/week MeHg: 1.3 µg/kg bw/week	[4]
iAs	BMDL	0.06 µg/kg bw/day	[34]
Pb	BMDL	0.50 µg/kg bw/day ^a^ 0.63 µg/kg bw/day ^b^ 1.50 µg/kg bw/day ^c^	[3]

^a^ BMDL for children; ^b^ BMDL for adults, based on nephrotoxic effect; ^c^ BMDL for adults, based on cardiovascular effects.

**Table 2:** List of food additives analyzed and their chemical formulas according to Commission Regulation 231/2012 (NS = not specified) [12]. Functional classes according to the Codex Alimentarius Guideline of 2026 [36].

E Number	Name	Chemical Formula ^a^	Functional Classes
E 120	Carminic acid	C_22_H_20_O_13_	Color
E 170	Calcium carbonate	CaCO_3_	Acidity regulator Anticaking agent Color Firming agent Flour treatment agent Stabilizer
E 300	Ascorbic acid	C_6_H_8_O_6_	Acidity regulator Antioxidant Flour treatment agent Sequestrant
E 301	Sodium ascorbate	C_6_H_7_O_6_Na	Antioxidant Flour treatment agent
E 330	Citric acid	C_6_H_8_O_7_	Acidity regulator Antioxidant Color retention agent Sequestrant
E 401	Sodium alginate	(C_6_H_7_NaO_6_)*_n_*	Bulking agent Carrier Emulsifier Foaming agent Gelling agent Glazing agent Humectant Sequestrant Stabilizer Thickener
E 407	Carrageenan	NS ^a^	Bulking agent Carrier Emulsifier Gelling agent Glazing agent Humectant Stabilizer Thickener
E 410	Locust bean gum	NS ^a^	Emulsifier Gelling agent Stabilizer Thickener
E 412	Guar gum	NS ^a^	Emulsifier Stabilizer Thickener
E 415	Xanthan gum	NS ^a^	Emulsifier Foaming agent Stabilizer Thickener
E 422	Glycerol	C_3_H_8_O_3_	Humectant Thickener
E 440	Pectin	NS ^a^	Emulsifier Gelling agent Glazing agent Stabilizer Thickener
E 461	Methyl cellulose	C_6_H_7_O_2_(OR_1_)(OR_2_)(OR_3_) where R_1_, R_2_, R_3_ each may be one of the following: -H -CH_3_ or -CH_2_CH_3_	Bulking agent Emulsifier Glazing agent Stabilizer Thickener
E 500	Sodium carbonates	Na_2_CO_3_·nH_2_O (*n* = 0, 1 or 10) NaHCO_3_ Na_2_CO_3_·NaHCO_3_·2H_2_O	Acidity regulator Anticaking agent Emulsifying salt Raising agent Stabilizer Thickener
E 508	Potassium chloride	KCl	Firming agent Flavor enhancer Stabilizer Thickener
E 509	Calcium chloride	CaCl_2_·nH_2_O (*n* = 0, 2 or 6)	Firming agent Stabilizer Thickener
E 516	Calcium sulphate	CaSO_4_·nH_2_O (*n* = 0 or 2)	Acidity regulator Color Firming agent Flour treatment agent Sequestrant Stabilizer

^a^ NS = not specified.

**Table 3:** Performance parameters of the methods (LOQ = limit of quantification, RSDrw = pooled reproducibility relative standard deviation, U = expanded measurement uncertainty, k = coverage factor, HMI = high matrix introduction).

Group	ICP-MS Measurement Mode	Element	LOQ (µg/kg)	RSD_rw_ (%)	Trueness Recovery (%)	U (k = 2) (%)
All		Hg	0.6	4.3	90–99	25.8
		iAs	4.0	2.1	83–98	21.0
Gums	General	As	3.0	4.2	100–110	19.0
		Cd	2.6	6.5	98–107	20.2
		Pb	13.2	5.4	101–109	19.0
Polysaccharides	General	As	3.0	5.0	99–109	18.0
		Cd	2.6	6.0	98–104	14.8
		Pb	13.2	6.9	94–102	17.6
Celluloses	General	As	3.0	4.7	102–107	17.6
		Cd	2.6	5.4	103–108	19.4
		Pb	13.2	5.3	102–105	16.0
Other organic molecules	General	As	3.0	3.9	103–105	13.8
		Cd	2.6	5.2	104–106	16.4
		Pb	13.2	8.4	107–108	26.6
Minerals Calcium carbonate	HMI	As	18.9	7.6	99–105	20.2
		Cd	2.5	11.1	98–99	28.6
		Pb	17.2	6.5	101–106	18.2
Minerals Sodium carbonates	HMI	As	18.9	7.4	94–105	21.4
		Cd	2.5	6.8	92–102	19.8
		Pb	17.2	8.4	90–102	25.2
Minerals Potassium chloride	HMI	As	18.9	3.0	101–107	12.4
		Cd	2.5	6.5	100–110	18.8
		Pb	17.2	3.8	99–109	15.8
Minerals Calcium chloride	HMI	As	18.9	5.8	95–104	16.6
		Cd	2.5	6.5	94–105	18.4
		Pb	17.2	3.2	92–98	13.2
Minerals Calcium sulphate	HMI	As	33.3	12.2	98–108	33.3
		Cd	50.6	8.6	97–107	23.3
		Pb	145.3	5.9	104–106	18.9

**Table 4:** Median (minimum-maximum) concentrations (µg/kg) and specification limits (SL; µg/kg; laid down in Commission Regulation (EU) No 231/2012) [12] of As, Cd, Pb and Hg in selected food additives.

E Number	n	As	iAs	Cd	Pb	Hg
Carminic acid	10	59.0 (<3–149)	98 (n = 1)	5.5 (<2.6–33.0)	234 (<13.2–873)	16.2 (0.8–18.7) (n = 4)
SL ^a^		1000		100	1500	500
Calcium carbonate	5	107 (56.5–252)		207 (24.6–471)	172 (147–400)	0.9 (<0.6–1.5) (n = 4)
SL ^a^		3000		1000	3000	NA
Ascorbic acid	6	<3.0 (<3.0–<3.0)		<2.6 (<2.6–<2.6)	<13.2 (<13.2–245)	<0.6 (<0.6–<0.6)
SL ^a^		3000		NA	2000	1000
Sodium ascorbate	5	<3.0 (<3.0–<3.0)		<2.6 (<2.6–<2.6)	<13.2 (<13.2–<13.2)	<0.6 (<0.6–<0.6)
SL ^a^		3000		NA	2000	1000
Citric acid	12	6.2 (<3.0–46.6)		<2.6 (<2.6–<2.6)	<13.2 (<13.2–<13.2)	<0.6 (<0.6–<0.6) (n = 3)
SL ^a^		1000		NA	500	1000
Sodium alginate	5	474 (340–685)	6.8 (4.0–18.0)	5.4 (<2.6–9.3)	187 (117–2147)	<0.6 (<0.6–7.4)
SL ^a^		3000		1000	5000	1000
Carrageenan	5	354 (54.1–1516)	152 (112–155) (n = 3)	110 (30.6–623.1)	186 (75.9–282)	1.6 (0.8–1.7)
SL ^a^		3000		2000	5000	1000
Locust bean gum	5	4.9 (<3.0–31)		4.1 (<2.6–5.3)	109 (<13.2–230)	<0.6 (<0.6–0.9) (n = 4)
SL ^a^		3000		1000	2000	1000
Guar gum	7	7.0 (4.1–13.1)		6.9 (5.4–8.1)	<13.2 (<13.2–28.3)	<0.6 (<0.6–<0.6) (n = 3)
SL ^a^		3000		1000	2000	1000
Xanthan gum	5	9.0 (5.5–33.0)		3.2 (<2.6–8.0)	<13.2 (<13.2–85)	1.6 (<0.6–1.9) (n = 3)
SL ^a^		NA		NA	2000	NA
Glycerol	5	3.6 (<3.0–9.0)		<2.6 (<2.6–<2.6)	<13.2 (<13.2–<13.2)	<0.6 (<0.6–<0.6) (n = 3)
SL ^a^		100		100	100	100
Pectin	7	11.1 (<3.0–13.7)		<2.6 (<2.6–<2.6)	204 (<13.2–1019)	<0.6 (<0.6–0.6)
SL ^a^		3000		1000	5000	1000
Methyl cellulose	5	<3.0 (<3.0–<3.0)		<2.6 (<2.6–<2.6)	<13.2 (<13.2–26.3)	<0.6 (<0.6–0.8) (n = 3)
SL ^a^		3000		1000	2000	1000
Sodium carbonates	7	<18.9 (<18.9–<18.9)		<2.5 (<2.5–16.4)	<17.2 (<17.2–84.6)	<0.6 (<0.6–<0.6) (n = 2)
SL ^a^		3000		NA	2000	1000
Potassium chloride	5	<18.9 (<18.9–<18.9)		<2.5 (<2.5–6.2)	119 (28.7–235)	<0.6 (<0.6–<0.6) (n = 3)
SL ^a^		3000		1000	2000	1000
Calcium chloride	5	<18.9 (<18.9–<18.9)		10.7 (3.1–152)	1159 (292–2252)	<0.6 (<0.6–0.7) (n = 4)
SL ^a^		3000		NA	2000	1000
Calcium sulphate	5	355 (277–575)		<50.6 (<50.6–<50.6)	<145 (<145–246)	0.7 (0.7–0.8) (n = 3)
SL ^a^		3000		NA	2000	1000

^a^ SL = specification limits (µg/kg) as published on 27 October 2024 and used in this study, NA = not available which means that no specification limit has been set. Please note that some figures have been revised since then [37,38].

**Table 5:** Estimated aggregated exposure to Pb and iAs (expressed in µg/kg bw/day) due to the combined intake of different food additives.

	Pb Exposure			iAs Exposure		
Age Group	Mean	Median	P95	Mean	Median	P95
	µg/kg bw/day			µg/kg bw/day		
Children	0.115	0.101	0.229	0.029	0.028	0.045
Adolescents	0.052	0.047	0.097	0.011	0.011	0.018
Adults	0.038	0.033	0.074	0.009	0.009	0.015
Elderly	0.033	0.030	0.060	0.009	0.009	0.014

**Table 6:** Estimated aggregated exposure to Cd and Hg (expressed in µg/kg bw/week) due to the combined intake of different food additives.

	Cd Exposure			Hg Exposure		
Age Group	Mean	Median	P95	Mean	Median	P95
	µg/kg bw/week			µg/kg bw/week		
Children	0.180	0.147	0.426	0.0027	0.0025	0.0050
Adolescents	0.074	0.064	0.160	0.0010	0.0010	0.0017
Adults	0.054	0.046	0.124	0.0008	0.0008	0.0014
Elderly	0.056	0.048	0.127	0.0008	0.0008	0.0014

## Data Availability

The data generated and the R code files are publicly available on Zenodo: https://doi.org/10.5281/zenodo.22654960.

## Supplementary Materials

The following supporting information can be downloaded at: https://www.mdpi.com/article/10.3390/foods15203734/s1, Table S1: Distribution models applied to food additive intake data. Mean and 95th percentile (P95) intake values are specific to Belgian population groups and are derived from the respective food additive re-evaluations by EFSA, available in the EFSA Journal. Table S2: Distribution models applied to lead impurity concentration data in food additives. The models were selected based on expert judgement and are not necessarily the best-fit models. Table S3: Distribution models applied to cadmium impurity concentration data in food additives Table S4: Distribution models applied to arsenic impurity concentration data in food additives. Table S5: Distribution models applied to mercury impurity concentration data in food additives. Table S6: Lead exposure via food additive consumption (µg/kg bw/day) per age group. Table S7: Arsenic exposure via food additive consumption (µg/kg bw/day) per age group. Table S8: Cadmium exposure via food additive consumption (µg/kg bw/day) per age group. Table S9: Mercury exposure via food additive consumption (µg/kg bw/day) per age group.
