# Supplementary material for: Occurrence of and Dietary Exposure to Toxic Element Impurities in Food Additives: A Probabilistic Exposure Assessment for the Belgian Population
Source: Foods. 2026 Oct 20;15(20):3734. doi: 10.3390/foods15203734 (PMC13704562; doi:10.3390/foods15203734)
Supplement: Supplementary file 1 [file foods-15-03734-s001.zip › foods-4585229-supplementary.pdf]

foods

MDPI

Supplementary Material

Table S1. Distribution models applied to food additive intake data. Mean and 95th percentile (P95) intake values are specific to Belgian population groups and are derived from the respective food additive re-evaluations by EFSA, available in the EFSA Journal.

FA	Age group	Mean/P95 intake (mg/kg bw/d)	Reference	Distribution model[b, c]
E 120	Children	0.2/0.4	[11]	Gamma
	Adolescents	0.1/0.3		
	Adults	0.1/0.3		
	Elderly	0.1/0.3		
E 170	Children	35/69	[54]	Normal, truncated
	Adolescents	20/42		
	Adults	11/27		
	Elderly	11/32		
E 401	Children	68/121	[15]	Normal, truncated
	Adolescents	19/37		
	Adults	16/34		
	Elderly	16/31		
E 407	Children	58/110	[17]	Normal, truncated
	Adolescents	16/30		
	Adults	14/27		
	Elderly	14/27		
E 410	Children	106/190	[28]	Normal, truncated
	Adolescents	36/68		
	Adults	32/64		
	Elderly	33/60		
E 415	Children	30/64	[19]	Normal, truncated
	Adolescents	13/27		
	Adults	11/24		
	Elderly	13/26		
E 440	Children	78/179	[29]	Normal, truncated
	Adolescents	25/57		
	Adults	18/46		
	Elderly	12/35		
E 508 & E 509	Children	(15/35)[a]	[30]	Gamma
	Adolescents	15/35		
	Adults	9/24		
	Elderly	6/13		
E 516	Children	29/53	[31]	Normal, truncated
	Adolescents	11/20		
	Adults	9/18		
	Elderly	8/17		

FA	Age group	Mean/P95 intake (mg/kg bw/d)	Reference	Distribution model[b, c]
E 412	Children	50/90	[16]	Normal, truncated
	Adolescents	17/35		
	Adults	16/32		
	Elderly	18/35		
E 422	Children	240/402	[10]	Normal, truncated
	Adolescents	125/227		
	Adults	95/198		
	Elderly	71/130		
E 461	Children	52/97	[49]	Normal, truncated
	Adolescents	30/58		
	Adults	21/47		
	Elderly	15/29		

[a] Mean and P95 not available, values for adolescents are applied to children; [b] The models were selected based on expert judgement and are not necessarily the best-fit models; [c] Truncation is done at zero and twice the P95 intake.

Table S2. Distribution models applied to lead impurity concentration data in food additives. The models were selected based on expert judgement and are not necessarily the best-fit models.

**FA**	**Pb range (mg/kg)**	**Distribution model**[a]	**Truncated**[b]
E 120	0-0.873	Gamma, truncated + 10% zero values	Min = 0.0044 mg/kg<br>Max = 1.3 mg/kg
E 170	0.147-0.4	Log-normal, truncated	Min = 0.075 mg/kg<br>Max = 0.60 mg/kg
E 401	0.117-2.15	Log-normal, truncated	Min = 0.059 mg/kg<br>Max = 3.2 mg/kg
E 407	0.076-0.282	Normal, truncated	Min = 0.038 mg/kg<br>Max = 0.42 mg/kg
E 410	0.013-0.229	Normal, truncated	Min = 0.0065 mg/kg<br>Max = 0.34 mg/kg
E 412	0.005-0.028	Normal, truncated	Min = 0.0044<br>Max = 0.042
E 415	0-0.085	Log-normal, truncated + 20% zero values	Min = 0.0044 mg/kg<br>Max = 0.13 mg/kg
E 440	0.0059-1.019	Log-normal, truncated	Min = 0.0044 mg/kg<br>Max = 1.5 mg/kg
E 461	0-0.026	Normal, truncated, +20% zero values	Min = 0.0044<br>Max = 0.039
E 508	0.029-0.235	Normal, truncated	Min = 0.014 mg/kg<br>Max = 0.35 mg/kg
E 509	0.292-2.25	Normal, truncated	Min = 0.15 mg/kg<br>Max = 2.26[c] mg/kg
E 516	0.048-0.246	Log-normal, truncated	Min = 0.048 mg/kg<br>Max = 0.37 mg/kg

[a] The models were selected based on expert judgement and are not necessarily the best-fit models; [b] Truncation at LOD or 50% below the observed minimum concentration and at 50% above the observed maximum concentration. [c] The maximum value is set at the specification limit + the expanded measurement uncertainty.

Table S3. Distribution models applied to cadmium impurity concentration data in food additives.

FA	Cd range (mg/kg)	Distribution model[a]	Truncated[b]
E 120	0-0.033	Normal, truncated + 20% zero values	Min = 0 mg/kg<br>Max = 0.05 mg/kg
E 170	0.025-0.471	Normal, truncated	Min = 0.012 mg/kg<br>Max = 0.707 mg/kg
E 401	0.002-0.009	Normal, truncated	Min = 0.001 mg/kg<br>Max = 0.014 mg/kg
E 407	0.031-0.623	Lognormal, truncated	Min = 0.015 mg/kg<br>Max = 0.935 mg/kg
E 410	0.002-0.005	Uniform	Min = 0.001 mg/kg<br>Max = 0.008 mg/kg
E 412	0.005-0.008	Uniform	Min = 0.003 mg/kg<br>Max = 0.012 mg/kg
E 415	0.001-0.008	Normal, truncated	Min = 0.0005 mg/kg<br>Max = 0.012 mg/kg
E 440	0-0.521	Lognormal, truncated + 14% zero values	Min = 0 mg/kg<br>Max = 0.782 mg/kg
E 509	0.003-0.152	Lognormal, truncated	Min = 0.002 mg/kg<br>Max = 0.228 mg/kg

[a] The models were selected based on expert judgement and are not necessarily the best-fit models; [b] Truncation at LOD or 50% below the observed minimum concentration and at 50% above the observed maximum concentration.

Table S4. Distribution models applied to arsenic impurity concentration data in food additives

FA	As range (mg/kg)	Distribution model[a]	Truncated[b]
E 120	0.002-0.149	Normal, truncated	Min = 0.0009 mg/kg<br>Max = 0.223 mg/kg
E 170	0.056-0.252	Lognormal, truncated	Min = 0.028 mg/kg<br>Max = 0.378 mg/kg
E 401	0.004-0.018	Lognormal, truncated	Min = 0.002 mg/kg<br>Max = 0.027 mg/kg
E 407	0.054-0.155	Normal, truncated	Min = 0.027 mg/kg<br>Max = 0.233 mg/kg
E 410	0.002-0.030	Normal, truncated	Min = 0.001 mg/kg<br>Max = 0.046 mg/kg
E 412	0.004-0.013	Normal, truncated	Min = 0.002 mg/kg<br>Max = 0.020 mg/kg
E 415	0.005-0.033	Normal, truncated	Min = 0.003 mg/kg<br>Max = 0.049 mg/kg
E 440	0.002-0.014	Normal, truncated	Min = 0.0009 mg/kg<br>Max = 0.021 mg/kg
E 422	0.003-0.009	Lognormal, truncated + 20% zero values	Min = 0 mg/kg<br>Max = 0.013 mg/kg
E 516	0.277-0.575	Normal, truncated	Min = 0.138 mg/kg<br>Max = 0.862 mg/kg

[a] The models were selected based on expert judgement and are not necessarily the best-fit models; [b] Truncation at LOD or 50% below the observed minimum concentration and at 50% above the observed maximum concentration.

Table S5. Distribution models applied to mercury impurity concentration data in food additives.

FA	Hg range (mg/kg)	Distribution model[a]	Truncated[b]
E 120	0.00085-0.019	Normal, truncated	Min = 0.00042 mg/kg<br>Max = 0.028 mg/kg
E 170	0.00059-0.0015	Normal, truncated	Min = 0.00029 mg/kg<br>Max = 0.0023 mg/kg
E 401	0.00030-0.007	Lognormal, truncated	Min = 0.00018 mg/kg<br>Max = 0.011 mg/kg
E 407	0.00085-0.002	Normal, truncated	Min = 0.00042 mg/kg<br>Max = 0.0025 mg/kg
E 410	0.00024-0.00089	Lognormal, truncated	Min = 0.00018 mg/kg<br>Max = 0.0013 mg/kg
E 415	0.00052-0.002	Normal, truncated	Min = 0.00026 mg/kg<br>Max = 0.0028 mg/kg
E 461	0.00045-0.00078	Lognormal, truncated	Min = 0.00023 mg/kg<br>Max = 0.0012 mg/kg
E 509	0.00045-0.00068	Normal, truncated	Min = 0.00023 mg/kg<br>Max = 0.0010 mg/kg
E 516	0.00070-0.00084	Normal, truncated	Min = 0.00035 mg/kg<br>Max = 0.0013 mg/kg

[a] The models were selected based on expert judgement and are not necessarily the best-fit models; [b] Truncation at LOD or 50% below the observed minimum concentration and at 50% above the observed maximum concentration.

Table S6. Lead exposure via food additive consumption (μg/kg bw/day) per age group

Age group	Food additive	Mean	Median	P95
			μg/kg bw/day	
Children	E 120	0.00007	0.00004	0.00026
	E 170	0.00801	0.00702	0.01777
	E 401	0.03530	0.02085	0.11803
	E 407	0.01180	0.01037	0.02634
	E 410	0.01324	0.01108	0.03236
	E 412	0.00080	0.00071	0.00179
	E 415	0.00089	0.00048	0.00326
	E 440	0.01960	0.00698	0.08575
	E 461	0.00073	0.00065	0.00190
	E 508	0.00196	0.00144	0.00547
	E 509	0.01800	0.01364	0.04800
	E 516	0.00446	0.00386	0.01005
Adolescents	E 120	0.00004	0.00001	0.00016
	E 170	0.00470	0.00408	0.01068
	E 401	0.01021	0.00590	0.03431
	E 407	0.00325	0.00288	0.00725
	E 410	0.00475	0.00392	0.01185
	E 412	0.00028	0.00024	0.00066
	E 415	0.00037	0.00020	0.00138
	E 440	0.00618	0.00215	0.02657
	E 461	0.00043	0.00038	0.00115
	E 508	0.00199	0.00148	0.00551
	E 509	0.01830	0.01403	0.04898
	E 516	0.00171	0.00149	0.00384
Adults	E 120	0.00003	0.00001	0.00015
	E 170	0.00293	0.00251	0.00698

Age group	Food additive	Mean	Median	P95
		μg/kg bw/day		
	E 401	0.00890	0.00512	0.03037
	E 407	0.00289	0.00255	0.00652
	E 410	0.00421	0.00340	0.01061
	E 412	0.00027	0.00023	0.00065
	E 415	0.00033	0.00018	0.00119
	E 440	0.00477	0.00164	0.02118
	E 461	0.00032	0.00027	0.00088
	E 508	0.00116	0.00073	0.00368
	E 509	0.01057	0.00684	0.03282
	E 516	0.00144	0.00124	0.00335
Elderly	E 120	0.00004	0.00001	0.00015
	E 170	0.00336	0.00279	0.00839
	E 401	0.00878	0.00510	0.03018
	E 407	0.00283	0.00253	0.00622
	E 410	0.00415	0.00346	0.01043
	E 412	0.00030	0.00025	0.00069
	E 415	0.00036	0.00020	0.00130
	E 440	0.00372	0.00122	0.01645
	E 461	0.00022	0.00019	0.00056
	E 508	0.00080	0.00062	0.00207
	E 509	0.00736	0.00589	0.01843
	E 516	0.00136	0.00117	0.00316

Table S7. Arsenic exposure via food additive consumption (μg/kg bw/day) per age group

Age group	Food additive	Mean	Median	P95
		μg/kg bw/day		
Children	E 120	0.000023	0.000016	0.000070
	E 170	0.004565	0.003688	0.011368
	E 401	0.000660	0.000547	0.001569
	E 407	0.006867	0.006280	0.014505
	E 410	0.001845	0.001545	0.004520
	E 412	0.000429	0.000396	0.000884
	E 415	0.000644	0.000526	0.001629
	E 422	0.001118	0.001047	0.002718
	E 440	0.000854	0.000705	0.002092
	E 516	0.011929	0.011153	0.024042
Adolescents	E 120	0.000011	0.000009	0.000029
	E 170	0.002674	0.002105	0.006958
	E 401	0.000190	0.000153	0.000481
	E 407	0.001896	0.001720	0.004082
	E 410	0.000635	0.000510	0.001627
	E 412	0.000155	0.000137	0.000353
	E 415	0.000274	0.000223	0.000708
	E 422	0.000597	0.000543	0.001524
	E 440	0.000273	0.000230	0.000663
	E 516	0.004552	0.004219	0.009099
Adults	E 120	0.000006	0.000003	0.000020
	E 170	0.001682	0.001299	0.004421
	E 401	0.000169	0.000136	0.000430
	E 407	0.001675	0.001509	0.003585
	E 410	0.000575	0.000464	0.001466
	E 412	0.000144	0.000128	0.000320

Age group	Food additive	Mean	Median	P95
			µg/kg bw/day	
	E 415	0.000241	0.000197	0.000605
	E 422	0.000481	0.000404	0.001296
	E 440	0.000215	0.000177	0.000542
	E 516	0.003936	0.003602	0.008321
Elderly	E 120	0.000006	0.000003	0.000020
	E 170	0.001898	0.001444	0.005153
	E 401	0.000162	0.000130	0.000412
	E 407	0.001669	0.001495	0.003600
	E 410	0.000576	0.000475	0.001430
	E 412	0.000159	0.000143	0.000350
	E 415	0.000264	0.000217	0.000652
	E 422	0.000337	0.000303	0.000852
	E 440	0.000160	0.000130	0.000407
	E 516	0.003653	0.003362	0.007612

Table S8. Cadmium exposure via food additive consumption (µg/kg bw/day) per age group

Age group	Food additive	Mean	Median	P95
		µg/kg bw/day		
Children	E 120	0.000003	0.000002	0.000008
	E 170	0.009104	0.007469	0.022751
	E 401	0.000399	0.000358	0.000851
	E 407	0.011986	0.006769	0.041741
	E 410	0.000487	0.000424	0.001093
	E 412	0.000372	0.000327	0.000799
	E 415	0.000162	0.000132	0.000408
	E 440	0.002739	0.000418	0.012724
	E 509	0.000425	0.000169	0.001719
Adolescents	E 120	0.000001	0.000001	0.000005
	E 170	0.005380	0.004308	0.013733
	E 401	0.000115	0.000100	0.000259
	E 407	0.003318	0.001821	0.011585
	E 410	0.000170	0.000146	0.000401
	E 412	0.000133	0.000114	0.000307
	E 415	0.000069	0.000056	0.000174
	E 440	0.000902	0.000137	0.004282
	E 509	0.000428	0.000167	0.001750
Adults	E 120	0.000001	0.000001	0.000005
	E 170	0.003410	0.002697	0.009168
	E 401	0.000101	0.000088	0.000232
	E 407	0.002942	0.001644	0.010486
	E 410	0.000153	0.000127	0.000366
	E 412	0.000124	0.000107	0.000284
	E 415	0.000060	0.000048	0.000154
	E 440	0.000723	0.000103	0.003464

Age group	Food additive	Mean	Median	P95
			μg/kg bw/day	
	E 509	0.000249	0.000087	0.001045
Elderly	E 120	0.000001	0.000001	0.000005
	E 170	0.003905	0.002974	0.010665
	E 401	0.000098	0.000087	0.000219
	E 407	0.002945	0.001632	0.010441
	E 410	0.000149	0.000128	0.000342
	E 412	0.000138	0.000120	0.000314
	E 415	0.000066	0.000054	0.000164
	E 440	0.000509	0.000074	0.002376
	E 509	0.000176	0.000073	0.000723

Table S9. Mercury exposure via food additive consumption (µg/kg bw/day) per age group

Age group	Food additive	Mean	Median	P95
			µg/kg bw/day	
Children	E 120	0.000005	0.000004	0.000016
	E 170	0.000036	0.000032	0.000082
	E 401	0.000117	0.000063	0.000419
	E 407	0.000079	0.000074	0.000157
	E 410	0.000049	0.000042	0.000115
	E 415	0.000046	0.000040	0.000109
	E 461	0.000030	0.000028	0.000062
	E 509	0.000008	0.000007	0.000019
	E 516	0.000022	0.000022	0.000041
Adolescents	E 120	0.000003	0.000002	0.000006
	E 170	0.000021	0.000019	0.000050
	E 401	0.000034	0.000018	0.000120
	E 407	0.000022	0.000021	0.000045
	E 410	0.000017	0.000014	0.000040
	E 415	0.000019	0.000017	0.000045
	E 461	0.000018	0.000017	0.000036
	E 509	0.000008	0.000007	0.000019
	E 516	0.000008	0.000008	0.000015
Adults	E 120	0.000001	0.000001	0.000004
	E 170	0.000013	0.000011	0.000033
	E 401	0.000029	0.000016	0.000107
	E 407	0.000019	0.000018	0.000039
	E 410	0.000015	0.000013	0.000037
	E 415	0.000017	0.000015	0.000040
	E 461	0.000013	0.000012	0.000029
	E 509	0.000005	0.000003	0.000013

Age group	Food additive	Mean	Median	P95
			μg/kg bw/day	
	E 516	0.000007	0.000007	0.000014
Elderly	E 120	0.000001	0.000001	0.000004
	E 170	0.000015	0.000012	0.000038
	E 401	0.000029	0.000016	0.000105
	E 407	0.000019	0.000018	0.000038
	E 410	0.000015	0.000012	0.000035
	E 415	0.000019	0.000016	0.000043
	E 461	0.000009	0.000008	0.000018
	E 509	0.000003	0.000003	0.000007
	E 516	0.000007	0.000007	0.000013

References

11. EFSA Scientific Opinion on the Re-Evaluation of Cochineal, Carminic Acid, Carmines (E 120) as a Food Additive. *EFSA J.* **2015**, *13*, 4288, doi:10.2903/j.efsa.2015.4288.
54. EFSA Re-Evaluation of Calcium Carbonate (E 170) as a Food Additive in Foods for Infants below 16 Weeks of Age and Follow-up of Its Re-Evaluation as Food Additive for Uses in Foods for All Population Groups. *EFSA J.* **2023**, *21*, e08106, doi:10.2903/j.efsa.2023.8106.
15. EFSA Re-Evaluation of Alginic Acid and Its Sodium, Potassium, Ammonium and Calcium Salts (E 400–E 404) as Food Additives. *EFSA J.* **2017**, *15*, e05049, doi:10.2903/j.efsa.2017.5049.
17. EFSA Re-Evaluation of Carrageenan (E 407) and Processed Eucheuma Seaweed (E 407a) as Food Additives. *EFSA J.* **2018**, *16*, e05238, doi:10.2903/j.efsa.2018.5238.
28. EFSA Re-Evaluation of Locust Bean Gum (E 410) as a Food Additive. *EFSA J.* **2017**, *15*, e04646, doi:10.2903/j.efsa.2017.4646.
19. EFSA Re-Evaluation of Xanthan Gum (E 415) as a Food Additive. *EFSA J.* **2017**, *15*, e04909, doi:10.2903/j.efsa.2017.4909.
29. EFSA Re-Evaluation of Pectin (E 440i) and Amidated Pectin (E 440ii) as Food Additives. *EFSA J.* **2017**, *15*, e04866, doi:10.2903/j.efsa.2017.4866.
30. EFSA Re-Evaluation of Hydrochloric Acid (E 507), Potassium Chloride (E 508), Calcium Chloride (E 509) and Magnesium Chloride (E 511) as Food Additives. *EFSA J.* **2019**, *17*, e05751, doi:10.2903/j.efsa.2019.5751.
31. EFSA Re-Evaluation of Sulphuric Acid and Its Sodium, Potassium, Calcium and Ammonium Salts (E 513, 514 (i), 514 (Ii), 515 (i), 515 (Ii), 516 and 517) as Food Additive. *EFSA J.* **2019**, *17*, e05868, doi:10.2903/j.efsa.2019.5868.
16. EFSA Re-Evaluation of Guar Gum (E 412) as a Food Additive. *EFSA J.* **2017**, *15*, e04669, doi:10.2903/j.efsa.2017.4669.
10. EFSA Re-Evaluation of Glycerol (E 422) as a Food Additive. *EFSA J.* **2017**, *15*, e04720, doi:10.2903/j.efsa.2017.4720.
49 EFSA Re-Evaluation of Celluloses E 460(i), E 460(Ii), E 461, E 462, E 463, E 464, E 465, E 466, E 468 and E 469 as Food Additives. *EFSA J.* **2018**, *16*, e05047, doi:10.2903/j.efsa.2018.5047.
